# Hypercapnia in the critically ill: insights from the bench to the bedside

**DOI:** 10.1098/rsfs.2020.0032

**Published:** 2021-02-12

**Authors:** Claire Masterson, Shahd Horie, Sean D. McCarthy, Hector Gonzalez, Declan Byrnes, Jack Brady, Juan Fandiño, John G. Laffey, Daniel O'Toole

**Affiliations:** Anaesthesia, School of Medicine and Regenerative Medicine Institute, National University of Ireland, Galway, Ireland

**Keywords:** carbon dioxide, hypercapnia, hypercapnic acidosis, inflammation, critical care, sepsis

## Abstract

Carbon dioxide (CO_2_) has long been considered, at best, a waste by-product of metabolism, and at worst, a toxic molecule with serious health consequences if physiological concentration is dysregulated. However, clinical observations have revealed that ‘permissive’ hypercapnia, the deliberate allowance of respiratory produced CO_2_ to remain in the patient, can have anti-inflammatory effects that may be beneficial in certain circumstances. In parallel, studies at the cell level have demonstrated the profound effect of CO_2_ on multiple diverse signalling pathways, be it the effect from CO_2_ itself specifically or from the associated acidosis it generates. At the whole organism level, it now appears likely that there are many biological sensing systems designed to respond to CO_2_ concentration and tailor respiratory and other responses to atmospheric or local levels. Animal models have been widely employed to study the changes in CO_2_ levels in various disease states and also to what extent permissive or even directly delivered CO_2_ can affect patient outcome. These findings have been advanced to the bedside at the same time that further clinical observations have been elucidated at the cell and animal level. Here we present a synopsis of the current understanding of how CO_2_ affects mammalian biological systems, with a particular emphasis on inflammatory pathways and diseases such as lung specific or systemic sepsis. We also explore some future directions and possibilities, such as direct control of blood CO_2_ levels, that could lead to improved clinical care in the future.

## 1. Introduction

While suspected for several years beforehand, Scottish chemist Joseph Black (1728–1799) first proved that carbon dioxide (CO_2_) was present in exhaled gas, and this led to many years of CO_2_ being considered solely a waste and dangerous by-product to be removed as efficiently as possible from biological systems during respiration. More recently, however, it has come to be recognized that CO_2_ has many profound effects on biological systems which are not exclusively deleterious. Investigations have been two pronged, with molecular approaches studying protein and other compound interactions with CO_2_, with specific sensing mechanisms previously reviewed [1], while administration or allowing accumulation of CO_2_ in whole animals or patients has given us both mechanistic insights and a window on therapeutic possibilities. In this review, we focus on acute inflammatory processes, particularly in the context of the critically ill patient, and how exploiting the rapidly expanding knowledge base regarding CO_2_ interactions with biological processes may ultimately lead to preservation of life rather than the originally described toxicity.

## 2. *In vitro* studies and advances

Cell culture-based studies are critical to understanding how hypercapnia (HC) and its associated acidosis (HCA) may affect clinical outcomes in patients receiving mechanical ventilation for acute respiratory distress syndrome (ARDS). Several cell culture-based findings have described the effects of HC on pulmonary cells; however, few have unravelled the mechanism by which these effects occur. A 2017 review by Shigemura *et al*. described the effects of HC on alveolar epithelial function and repair [2]. An earlier review by Ijland *et al*. compiled many of the early cell culture evidence of HC on alveolar macrophages, alveolar type II epithelial cells and pulmonary artery endothelial cells [3]. We have previously shown that HCA inhibits pulmonary epithelial wound healing by reducing cell migration via a nuclear factor kappa B (NFκB)-dependent mechanism [4]. In accordance with this, others reported HC to inhibit LPS-induced expression of interleukin (IL)-6 and tumour necrosis factor (TNF)-α in macrophages, fibroblasts and alveolar epithelial cells [5–7]. On the other hand, Wang *et al*. reported that HC did not affect LPS-induced degradation of IκB*α*, nuclear translocation of RelA/p65, or activation of mitogen-activated protein kinases [7]. While conversely, Keogh *et al*. provide molecular insight into the NFκB pathway and implicated altered RelB/p100-dependent signalling in HC induced inflammatory signalling [8]. A further study by Casalino-Matsuda *et al*. described the effects of elevated CO_2_ on alveolar macrophages in an influenza A virus (IAV) model [9]. HC increased IAV replication and inhibited antiviral gene and protein expression in macrophages both *in vivo* and *in vitro* through activation of protein kinase B [9]. ‘Permissive’ hypercapnia has previously been shown to increase neutrophil adherence in both *in vitro* and *in vivo* models of pulmonary inflammation as well as to increase IL-8 expression [10]. A 2016 study by the authors of this review showed that HC prevented cyclic mechanical stretch-induced pulmonary epithelial inflammation, injury and death. Furthermore, it was found that inhibition of stretch-induced canonical NFκB activation via breakdown of the cytosolic inhibitor IκB*α* was mediated by a pH-dependent mechanism rather than via CO_2_ *per se* [11]. Similar findings were reported more recently using a micropuncture wound model on lung epithelial cells exposed to HC [12].

Other signalling pathways are implicated in the mechanism by which HC controls cell functions, with studies noting effects on nucleosome and lipid metabolism in human airway epithelial cells [5], caspase-7-induced downregulation of the microRNA-133a [13] and the oxygen sensing hypoxia inducible factor *α* pathway [14]. What is less clear is to what extent individual points on these signalling pathways are subject to direct influence by HC or HCA or are further downstream of actual points of sensitivity or specific sensing.

One recent study reported the effects of CO_2_ concentration on gene expression in primary normal human bronchial epithelial cells. Genes linked to the immune response and nucleosome assembly were largely downregulated, while lipid metabolism genes were mainly upregulated in the hypercapnic state [5]. Another recent study in primary human small airway epithelium and capillary endothelium cells supported these findings as HC was found to attenuate the inflammatory response and induce mitochondrial dysfunction. They also proposed that mesenchymal stromal cells (MSCs) may not be therapeutically beneficial in hypercapnic ARDS patients [15,16], although the study was limited by being solely focused on MSC potential for wound repair of lung epithelium.

Recently, there have been several attempts to further elucidate the mechanisms and pathways underlying how HC influences pulmonary cells. Bharat *et al*. [17] reported that HC delayed wound closure of both large airway and alveolar epithelial cell monolayers. The study proposed that impaired healing was due to inhibition of epithelial cell migration by suppression of Rac1-GTPase via upregulation of AMP-kinase, concomitant with inhibition of NFκB-mediated CXCL12 release [17]. Another recent study investigated the effect of elevated CO_2_ levels on endoplasmic reticulum folding of NaK-ATPase (NKA) [18]. NKA is a basolateral membrane sodium–potassium pump in polarized cells and is critical in the reabsorption of fluid from the alveolar space facilitating efficient gas exchange. The group documented that HC decreased NKA cell surface abundance and function by preventing correct assembly of the protein subunits. Furthermore, the study reported that boosting ATP production could rescue the high CO_2_-induced mitochondrial dysfunction possibly restoring alveolar epithelial barrier function in patients with ARDS and HC. Similarly, elevated CO_2_ levels were shown to promote activation of the ERK/AMPK/JNK axis in a human alveolar epithelial cell line resulting in epithelial sodium channel dysfunction [19]. Disruption of airway surface fluid homeostasis by HC has also been reported in a cystic fibrosis model of ion and fluid transport using Calu-3 human airway epithelial cells [20]. A recent *in vitro* study of pulmonary cancer cells reported that the hypercapnic microenvironment of the tumour may result in chemoresistance to some therapies [21]. These reports highlight the complex mechanisms of HC and support the need for further investigation in larger models to reveal both the negative and positive effects that may be exploited for therapy.

## 3. The effects of hypercapnia in preclinical models of acute lung injury

The effects of HC and HCA have been studied in a number of preclinical models relating to acute lung injury (ALI) (table 1). One model that is used quite frequently to assess the effects of HC on the lung is the ventilator-induced lung injury (VILI) model in which the alveoli are purposely overstretched to inflict lung injury as is frequently seen in the clinical setting following aggressive, but necessary, ventilation regimens. In this model, HC (FICO_2_ 0.12) has been found to display protective effects on the lung in a dose- and time-dependant manner [24] when compared to normal ventilation without any CO_2_. These protective effects consist of reducing microvascular permeability and preventing the build-up of excess protein content and haemoglobin in the bronchoalveolar lavage fluid (BAL) along with a decrease in alveolar fluid reabsorption [22,23,25]. HC (PaCO_2_ 80–100 mmHg) in a rabbit model of VILI was also reported to prevent the increased levels of nitrates in the perfusate and BAL [22] which had previously been linked to a deleterious effect in the lung in experimental endotoxemia [46]. Treatment with HCA in rats and rabbits (FICO_2_ 0.05 and PaCO_2_ 80–100 mmHg, respectively) subjected to VILI has shown improvements in lung compliance and oxygenation levels along with a decrease in histological lung damage [23,26].

**Table 1.** The therapeutic effects of HC in preclinical models of ALI.

model	intervention	effects	reference
*VILI*			
VILI in rabbits	HCA (PaCO_2_ 80–100 mmHg)	↓ BAL protein ↓ BAL haemoglobin ↓ NOx	Broccard *et al*. [22]
VILI in rabbits	HCA (PaCO_2_ 80–100 mmHg)	↓ lung wet:dry weight ratio ↓ BAL protein and cell count ↓ histologic injury score	Sinclair *et al*. [23]
VILI in mice	HC (FICO_2_ 0.12)	↓ microvascular leak ↓ histologic injury ↓ inflammatory cytokines ↓ COX-2 ↑ lung tissue nitrotyrosine	Peltekova *et al*. [24]
VILI in rats	permissive HC	↑ alveolar gas distribution ↓ alveolar fluid reabsorption ↑ intracellular Ca^2+^ in AECs	Briva *et al*. [25]
VILI in rats	HCA (FICO_2_ 0.05)	↑ oxygenation ↑ lung compliance ↓ lung histologic injury ↓ BAL IL-6, TNF-*α* and CINC-1 ↓ BAL neutrophil infiltration ↓ decrement of IκB*α* ↓ NFκB activation ↓ cell death	Contreras *et al*. [26]
VILI in mice	HC (FICO_2_ 0.12)	↑ compliance ↓ BAL protein ↓ p44/42 MAPK activation ↓ADAM17 ↓ shedding of TNFR1	Otulakowski *et al*. [27]
VILI in rats	moderate–severe HCA (80–150 mmHg)	↑ paO_2_/FiO_2_ ↓ lung injury score ↓ MPO activity ↓ BAL TNF-α and MIP-2 ↓ NFκB activation ↓ lung ICAM-1 expression	Yang *et al*. [28]
VILI in mice	HC (FICO_2_ 0.12)	↑ pulmonary α-tocopherol transfer protein	Otulakowski *et al*. [29]
VILI in rats	HC (FICO_2_ 0.5)	↑ compliance, ↓ MPO activity ↓ BAL cytokines ↓ cell counts ↓ BAL protein ↓ histological injury score ↑ efferent vagus nerve activity	Xia *et al*. [30]
*reperfusion*			
isolated buffer-perfused lung in rabbits	HCA (FICO_2_ 0.25)	↓ pulmonary microvascular permeability ↓ capillary and pulmonary artery pressures	Shibata *et al*. [31]
lung reperfusion in rabbits	HC (FICO_2_ 0.12)	↓ pulmonary oedema ↓ apoptosis ↑ oxygenation ↓ BAL TNF-α ↓ lung tissue 8-isoprostane ↓ nitrotyrosine	Laffey *et al*. [32]
ischaemia-reperfusion induced lung injury in rats	HCA (10% CO_2_)	↓ inflammatory response ↓ NFκB	Wu *et al*. [33]
ischaemia-reperfusion induced lung injury in rats	HCA (5% CO_2_)	↑ HO-1 ↓ IKK-NFκB signalling ↓ apoptosis ↓ pulmonary oedema ↓ BAL TNF-α and neutrophils ↓ lung injury score	Wu *et al*. [34]
ischaemia-reperfusion injury in rats	HCA (5% CO_2_)	↓ serum liver enzymes ↓ TNF-α ↑ IL-10 ↓ histological score ↓ apoptotic index ↓ NFκB activation	Yan *et al*. [35]
*local and systemic infection*			
LPS in rats	HCA (FICO_2_ 0.05) over 4–6 h of ventilation	prophylactic HCA ↑ arterial oxygenation ↑ lung compliance ↓ alveolar neutrophil infiltration therapeutic HCA ↑ arterial oxygenation ↑ lung compliance ↓ neutrophil infiltration ↓ histologic lung injury	Laffey *et al*. [36]
*E. coli* in rats	HCA (FICO 0.05) over 6 h of ventilation with/without antibiotic	HCA without antibiotic therapy ↓ peak airway pressure ↑ lung compliance HCA with antibiotic therapy ↓ histologic lung injury	Ni Chonghaile *et al*. [37]
severe *E. coli* in rats	HCA (FICO_2_ 0.05) over 6 h of ventilation with/without neutrophil depletion	HCA without neutrophil depletion ↓ airway pressure ↑ lung compliance ↑ lung oxygenation HCA with neutrophil depletion ↓ both physiological and histological injury	Ni Chonghaile *et al*. [38]
early and prolonged caecal ligation and puncture (CLP) injury in mice	HCA (FICO_2_ 0.05) over 3 h of ventilation HCA (8% CO_2_) over 96 h	HCA in early systemic sepsis ↓ hypotension ↓ lactate ↓ neutrophil infiltration ↓ lung wet/dry weight ratios HCA in prolonged systemic sepsis ↓ histologic lung injury	Costello *et al*. [39]
*P. pneumonia* in mice	HCA (10% CO_2_) for 3 days prior and 4 days post infection	↓ neutrophil phagocytosis ↑ mortality	Gates *et al*. [40]
*E. coli* in rats	HCA (FICO_2_ 0.05) over 4 h of ventilation	↓ lung injury ↓ NFκB	Masterson *et al*. [41]
LPS in mice	HC pre-treatment with 5% CO_2_ inhalation for 10 or 60 min	10 min HC pre-treatment ↓ pulmonary oedema ↓ lung injury score ↓ neutrophil sequestration ↓ oxidative stress ↓ TLR4 surface expression ↓ NFκB	Tang *et al*. [42]
*other ALI models*			
surfactant-depleted rabbits	low tidal ventilation (2–10 ml kg^−1^) and permissive hypercapnia (40–160 mmHg) over 6 h	↓ lung wet–dry weight ratios ↓ histological lung injury ↑ paO_2_	Fuchs *et al*. [43]
paraquat in rats	HC (FICO_2_ 0.05) buffered HC	both HC and buffered HC ↓ IL-6 ↓ IL-1β ↓ type III procollagen ↓ lung cell apoptosis ↓ kidney cell apoptosis	Nardelli *et al*. [44]
surfactant-depleted rabbits	normoventilation-normocapnia tidal volume 7.5 ml kg^−1^, PaCO_2_ 40 mmHg normoventilation-HC tidal volume 7.5 ml kg^−1^, PaCO_2_ 80 mmHg (using inspired CO_2_) hypoventilation-HC tidal volume 4.5 ml kg^−1^, PaCO_2_ 80 mmHg (using inspired CO_2_)	hypoventilation-HC ↑ paO_2_ ↓ lung wet–dry-weight ratio ↓ BAL protein ↓ histologic injury ↓ IL-8 ↓ lactate	Hummler *et al*. [45]

Numerous studies have also noted a reduction in the presence of inflammatory cytokines in the BAL, including TNF-α, cytokine-induced neutrophil chemoattractant-1 (CINC-1), macrophage-inflammatory protein 2 (MIP-2) and IL-6 [26,28]. One such mechanism that could explain the associated decrease in inflammatory cytokine production with HC is the associated suppression of the NFκB pathway, as CO_2_ can prevent p65 translocation [6,26–28] (p65/RelA is a key transcription factor in the canonical NFκB pathway). Contreras *et al*. also noted a decrease in the degradation of IκB*α*, another limiting step in the activation of NFκB [26]. Decreased IL-8 production, decreased epithelial injury and decreased cell death were noted by the authors and attributed to the effects of HC on NFκB activation [26]. Whole animal models have used –omics approaches to determine the effects of CO_2_ on gene expression, with modulation seen on various signalling pathways such as increased Wnt signalling in *Caenorhabditis elegans*, *Drosophila melanogaster* and various mouse tissues [47].

The study by Peltekova *et al*. observed a reduction in the major inducible prostanoid-generating enzyme COX-2, although the downstream effects of this were reported to be modest [24]. One observation of note was the increase of lung tissue nitrotyrosine at PaCO_2_ levels that were deemed protective [24]. Whether this observation indicates benefit or harm is not fully understood as it could be associated with the inhibition of pro-inflammatory or pro-apoptotic proteins and thus provide therapeutic benefit [48]. Alternatively, this could be linked to enhanced peroxynitrite formation and its associated detrimental effects, including lipid peroxidation, depletion of glutathione, inhibition of mitochondrial respiration and direct DNA damage [49].

A study by Xia *et al*. recently identified that HC (FICO_2_ 0.05) enhanced vagus nerve activity to produce therapeutic effects in the lung [30]. They found that performing a vagotomy abolished the protective effects that HC provided which consisted of reduced MPO activity, reduced BAL cytokines, protein and cell count, and reduced histologic injury scores [30]. Otulakowski *et al*. showed that HC (FICO_2_ 0.12) inhibits stretch-mediated activation of p44/42 MAPK signalling in alveolar epithelial cells which occurs through the inhibition of the metalloprotease ADAM17 [27]. Pharmacological blockade of ADAM17 was shown to decrease downstream activation of MAPK activation and attenuate VILI in a two hit mouse model [27]. The same authors more recently, through genome wide gene expression analysis, discovered that HC (FICO_2_ 0.12) upregulates the α-tocopherol transporter (a potent anti-oxidant) and linked its benefits to the inhibition of pathogenic chemoattractant synthesis [29]

Another frequently used animal model of HC in the lung is the ischaemia-reperfusion model relevant to lung transplantations and to ARDS (table 1). Reductions in pulmonary oedema, lung stiffness and histological lung injury have been observed in numerous ischaemia-reperfusion studies [35,50]. Apoptosis of lung epithelial cells has been shown to be attenuated by HC treatment in a rabbit model of ischaemia-reperfusion injury [32]. Additionally, the same group found that buffering CO_2_, thereby ostensibly preserving normal physiological pH, in the same injury had a detrimental effect on lung injury severity [51]. It has to be mentioned however that buffering is a contentious issue, with the traditional sodium bicarbonate buffer possibly even increasing cytoplasmic acidity levels where newer patient options do not [50]. Furthermore, there are data suggesting bicarbonate therapy slows resolution of diabetic ketoacidosis and also slows lactate clearance, and that the benefits of bicarbonate on haemodynamics are due to the osmolar load [52]. Using HCA on uninjured lungs was shown to display no adverse reactions on the lung microvascular system and when injury was induced, HCA prevented the increase in pulmonary microvascular permeability [31]. Wu *et al*., in their studies, found that haem oxygenase-1, which is an anti-oxidant enzyme that protects tissue from inflammation injury, was upregulated by HC and blockade of this enzyme reversed the beneficial effects of HC [34]. Attenuation of inflammatory NFκB pathway signalling has also been attributed to HC in a range of ischaemia-reperfusion models [32–35].

Pneumonia and systemic infections are common causes of ARDS. HC has been studied in a number of pathogenic ALI models with both beneficial and possibly detrimental effects being reported (table 1). In an LPS instillation rat ALI model, both prophylactic and therapeutic HCA (exposure post LPS instillation) was shown to improve lung oxygenation and compliance, and attenuate neutrophil infiltration and histologic lung injury [36]. Interestingly, the study by Tang *et al*. showed that pre-treatment with HC (FICO_2_ 0.05) for 10 but not 60 min attenuated lung injury and inflammation in a murine LPS model [42].

In an *E. coli* induced lung injury rat model, HCA attenuated airway pressure and improved lung compliance; however this time lung histologic injury was only improved when HCA was administered in combination with antibiotic therapy potentially highlighting that HCA effects can be affected by the type of infectious insult [37]. In a similar study by the same group, HCA exposure in a severe *E. coli* induced ALI rat model improved lung physiology and attenuated lung histologic injury following neutrophil depletion in the model, suggesting that the beneficial effects of HCA in this acute setting are independent of any effect on neutrophils. [38]. Another study revealed that the mechanism by which HCA reduced *E. coli* induced lung injury was through the inhibition of NFκB [41]. HCA has also been shown to be therapeutic in early and prolonged models of systemic sepsis [39].

By contrast, other studies have reported that HCA (FICO_2_ 0.05 over 6 h of ventilation) did not protect against *E. coli* induced lung injury in rats even with antibiotic therapy [53]. Another study by the same group showed that environmental HC (5%) for 48 h worsened lung injury induced by *E. coli* instillation with or without antibiotic therapy in spontaneously breathing animals [54]. HC was also observed to impair neutrophil phagocytosis and thus alter bacterial clearance [54]. An emerging paradigm here is patient self-induced lung injury (P-SILI), where intense breathing effort may instigate or exacerbate lung injury, and could lead to profoundly different outcomes in ventilated and spontaneously breathing animals (or patients), which might explain some of the increased injury seen [55–58]. Interestingly, another study of *E. coli* administration in spontaneously breathing rats showed that HC at 5% for 24 h enhanced inflammation in the lung but reduced inflammation in the spleen [59]. The study indicated that initial HC exposure without adequate ventilation may cause hypoventilation that can exacerbate lung injury independently of HC exposure thereafter [59]. This finding is further re-established as HC in the absence of injury or stress does not exert harmful or beneficial effects in the lung [59].

In a murine *Pseudomonas* pneumonia model, HC exposure, which was instigated 3 days before and continued for 4 days after infection, was shown to increase mortality and again inhibit neutrophil phagocytosis [40]. HC exposure in this instance was at 10% and may have been too high, as 5% is often deemed a superior therapeutic level, and a P-SILI effect with increased tidal volume may have contributed to the mortality seen in the HC group. Furthermore, the effects of HC were time dependent as inflammatory cytokines in the lung were inhibited at 7 h but not at 15 h post infection [40]. Finally, the effect on mortality was reversed when HC was terminated before or immediately after infection [40]. Interestingly, another study found that LPS or *E. coli* lung injury can be worsened with prolonged prior exposure to HC (8% for three days) as it causes renal buffering of the HCA, that ultimately increases injury susceptibility [60]. This finding is further confirmed in the study by Higgins *et al*., whereby buffered HC was shown to be therapeutically ineffective against lung injury caused by systemic sepsis [61].

CO_2_ has been shown to be beneficial in other models of ALI of aseptic aetiology. Both HC and buffered HC attenuated lung inflammation and cell death following paraquat-toxin induced ALI [44]. Overall, this suggests that the potent anti-inflammatory effects of HCA can decrease the host's ability to fight infection and the effects of HCA, whether therapeutic or detrimental, are likely to be time dependent [54]. Surfactant-depleted rats treated with differing low tidal volume ventilation and associated HC levels were protected against lung injury [43]. Therapeutic HC in the same model offered protection against lung injury only in combination with low tidal volume ventilation [45]. By contrast, Liu *et al*. observed that HC exposure enhanced the ALI associated with gastric acid instillation in rats, though the levels may have been too high at 12% CO_2_ exposure given a cumulative effect of CO_2_ and gastric acid would lead to a very low and possibly directly injurious pH [62].

## 4. The use of increased CO_2_ levels in therapeutic approaches in human patients

To avoid the incidence of VILI in the critically ill, the use of lung protective strategies (including lower tidal volumes) have been employed to mitigate the over-distension and resulting injury of the lung [63,64] which also results in permissive HC. The success of lower tidal volume lung ventilatory strategies has led to investigations as to whether the associated HC is involved in the improved outcomes seen. As mentioned previously, the outcomes of preclinical studies have varied, whereby HC has shown both beneficial and harmful effects [51,65]. Clinically, the major trials which have been completed using protective ventilation strategies have demonstrated the protective effects of lower tidal/minute ventilation strategies [64,66–69]. The incidence of HC had not been well understood until the LUNG SAFE large prospective cohort study (LUNG SAFE, NCT02010073). The results of this study indicated that around 20% of ARDS patients on the first day had PaCO_2_ levels indicating that they were hypercapnic (greater than or equal to 50 mmHg) which correlated with increasing ARDS severity [70]. A retrospective analysis of the Acute Respiratory Distress Syndrome Network (ARDSNet) or ARMA trial demonstrated a possible role of HC in reducing 28 day mortality in patients receiving higher tidal volume ventilation independent of ARDS severity [71]. In a secondary analysis of non-interventional cohort studies, the presence of HC in patients at the early stages of ARDS correlated with an increased mortality rate; however this was without any adjustments for ARDS severity, baseline values or pH levels in these patients [72,73]. A more recent prospective cohort study has revealed little to no effect of HC in ARDS [73]. The direct use of therapeutic HC rather than permissive HC has not been investigated clinically as of yet in ARDS patients. HC has demonstrated conflicting results over the years of experimental research into ARDS; however, there have been promising results from the treatment of other critical illnesses and conditions as described below. The mechanism by which HC is produced is likely to be important where inhaled CO_2_ will ensure that even overventilated areas of the lung will have alveolar HC, whereas with permissive HC, i.e. HC due to reduced minute ventilation, there may still be areas of the lung that have alveolar normocapnia or even hypocapnia [74]. Therefore, a review of the clinical data available will give a greater insight as to when HC may be used or should be avoided. A study at the onset of ARDS investigating inhaled CO_2_ and equal systemic HC by intentional hypoventilation would be useful in this regard.

## 5. Surgical procedures

Given the positive effects demonstrated in various *in vitro*, *in vivo*, and large cohort retrospective analyses of clinical trials the direct investigation of the effects of HC in various ailments has been brought forward to clinical trial. The use of protective ventilation (low VT 6 ml kg^−1^ predicted body weight) in pancreaticoduodenal surgery patients demonstrated a positive effect in terms of reducing lactate levels, atelectasis and hospital stay, while increasing oxygenation levels [75]. Permissive HC and associated acidosis further lowered lactate levels but did not have any additional benefits, nor did it cause any harm. In a further investigation of the benefits of HC during surgical procedures, HC use to circumvent prolonged recovery after inhaled general anaesthesia was investigated in clinical trials. Here mild levels of HC (end tidal CO_2_ concentration 50–60 mmHg) were permitted through a decrease in minute ventilation. The time to recovery of full cognitive function was reduced significantly in hypercapnic patients (NCT00708526, NCT01151267) and HC was deemed a feasible and effective method to speed up recovery from general anaesthesia induced by inhaled agents [76]. In a randomized clinical trial, HC has been investigated for use in the prevention of surgical wound infection without success, albeit without any indication of harm (NCT00273377) [77]. In patients undergoing lobectomy, the HC group (PaCO_2_ 60–70 mmHg) had lower inflammatory and tissue injury markers in serum and BAL, increased oxygenation index and decreased airway pressure [78]. These results indicate a lack of strong evidence for an additional benefit in the use of permissive HC in surgical patients. However, there have been little to no indications of harm when using mild to moderate levels of HC.

## 6. Effects on the respiratory system

The PHARLAP (Permissive Hypercapnia Alveolar Recruitment and Low Airway Pressure) trial implemented a low tidal volume mechanical ventilation strategy (tidal volume: 4–6 ml kg^−1^; pH > 7.15) compared with a control mechanical ventilation strategy (tidal volume: 6 ml kg^−1^) for the treatment of ARDS. The primary outcome was the number of ventilator free days (VFD) at day 28 post randomization. A total number of 340 patients had been projected for the PHARLAP study, but at the moment of termination 115 patients had been enrolled. Hodgson *et al*. [79] have reported that the PHARLAP ventilator strategy reduced the use of new hypoxaemic adjuvant therapies and was associated with increased adverse effects in the cardiovascular system (CVS). In a more recent clinical trial, it was hypothesized that permissive HC would decrease the minute ventilation requirement event in patients with an increased dead space and would facilitate weaning these patients from mechanical ventilation (NCT00357929). This study did not detail the levels of HC and was ultimately terminated because of an insufficient recruitment at *n* = 3. Several studies have demonstrated a beneficial effect of hypercapnia in preserving lung compliance [32,80], though whether the mechanism is direct or via influence on other disease and repair systems remains unexplored.

Clinical investigations of the effects of HC on the respiratory system include observations of muscular contractility and function. In ARDS patients, skeletal and respiratory muscle weakness leads to impaired recovery [81,82] and reduced quality of life. In spontaneously breathing patients, HC can induce diaphragmatic fatigue and reduce contractility [83,84]; however in a small cohort of critically ill patients HC was not demonstrated to be a factor influencing diaphragmatic or skeletal muscle wastage [85]. More recently, Korponay *et al*. [86] were able to demonstrate the role of HC in repressing skeletal muscle ribosomal gene expression, specifically the downregulation of 45S preRNA, compared to normocapnia patients. This study suggests a relation between chronic HC and dysfunctional protein anabolism highlighting some of the potentially detrimental effects of HC. Taken together these results demonstrate that the effects of HC on muscle function in the critically ill are as yet unclear.

## 7. Effects on the cardiovascular system

The effects of HC on the CVS are mediated both directly and indirectly via sympathetic nervous system activation. HCA can increase cardiac output and decrease total peripheral resistance [87,88], leading to an overall net increase in cardiac index [89,90]. Tissue oxygenation can be positively influenced by the use of HC as demonstrated in healthy anaesthetized subjects whereby tissue oxygenation increased linearly with increasing HC due to an increase in cardiac output and improvements in peripheral tissue perfusion [89]. Some of the possible mechanisms by which HC can increase the tissue oxygenation are the following: (1) an increase in microvascular vasodilatation that promotes oxygen delivery and tissue perfusion [91]; (2) an improvement of ventilation-perfusion (V/Q) matching by potentiating hypoxic pulmonary vasoconstriction [74,92,93]; (3) an increase in cardiac output that increases peripheral oxygen delivery [94]; and/or (4) the Bohr effect, a change in the oxyhaemoglobin dissociation curve to the right which eases oxygen release to the tissues [95].

The use of permissive HC in 15 thoracotomy patients from 2011 to 2013 examined effects on right ventricle systolic and diastolic functions during one-lung ventilation. Moderate HC (60–70 mmHg) increased cardiac output and cardiac index significantly in all participants and was tolerated (NCT02519517).

In surgical procedures where positioning directly influences oxygenation, HC has also been investigated. Permissive HC was induced to 45 mmHg PaCO_2_ in patients undergoing surgery in a reclined seated position which commonly results in an impaired haemodynamic stability at baseline. The results of this study demonstrated that mild HC prevented the reduction of cerebral oxygen saturation without changes in oxygenation index and haemodynamic parameters [96].

HCA increases pulmonary vascular resistance in a porcine model [97], which may be deleterious in certain situations. A study currently recruiting patients following mitral valve prolapse repair surgery (NCT02757573) will investigate right ventricular responses to mild HC induced by controlled ventilation. Moderate levels of HC (approx. 56 mmHg/7.5 kPa) will be used and tricuspid annular plane systolic excursion change and mean pulmonary artery pressure changes measured with the hypothesis that mild HC will induce elevated mean pulmonary arterial pressure which will be evidenced by changes in volume and function of the right ventricle as detected by echocardiography. Conversely, other studies have found that HCA dilated pulmonary circulation [98,99], indicating much further research is required in this area.

A recently published retrospective report has determined the effects of CO_2_ levels on the mortality rate of cardiac arrest patients [100]. Patients were categorized into hypocapnia, normocapnia, mild and severe HC groups. While mild HC was not associated with an improved outcome, it was not associated with increased mortality when compared to normocapnia. The length of time patients spent in normocapnia was associated with an improved outcome and a lower hospital mortality rate. In a similar study, data from over 16 500 patients who suffered a non-traumatic cardiac arrest collected over an 11 year period found that the HC group (not broken into mild or severe) had similar mortality rates to normocapnia but a greater rate of discharge indicative of an improved neurological outcome [101].

In summary, these studies have indicated that the cardiovascular effects of HC are generally well tolerated with effects that may be beneficial in specific clinical situations. This has led to the initiation of several clinical trials to further investigate these therapeutic effects.

## 8. Cerebral system

Similar to the effects on the CVS, HC also demonstrates a potent augmentation of the cerebral blood flow (CBF) [102]; however, the value of this effect is not clear as HC results in conflicting patterns of regional cerebral perfusion in healthy subjects [103]. Therefore, caution must be taken as intracranial pressure increases can be induced by HC due to changes in CBF [104] which is not recommended in head trauma patients. Intracranial physiology contains intricately linked physiologies with the role of tissue viscoelasticity remaining undiscovered until a study performed by Hetzer *et al*. [105], who determined that HC-induced vasodilation in healthy volunteers led to an increase in brain viscoelasticity. These changes caused by HC also increased whole brain stiffness and viscosity. Kreft *et al*. [106] showed that cerebral stiffening during HC was affected by vascular pressure and blood perfusion in the brain of healthy volunteers. Their HC induction is also consistent with animal work performed by Van Hulst *et al*. [107], showing that increased intracranial pressure is associated with HC.

The effect of HC on CBF and brain tissue oxygen saturation (S_ti_O_2_) after aneurysmal subarachnoid haemorrhage (SAH) was studied in six mechanically ventilated patients (NCT01799525). Respiratory rate and minute ventilation were reduced gradually in 15-minute intervals to reach a PaCO_2_ level of 40, 50 and 60 mmHg and then returned to baseline. It was observed that both CBF and S_ti_O_2_ were gradually increased until PaCO_2_ level of 60 mmHg and after returning to baseline ventilator setting, this increment was maintained before gradually returning to baseline parameters without rebound effect. None of the patients suffered a secondary cerebral infarction. It was concluded that HC causes a global increase of brain perfusion and oxygen supply and it is safe and feasible for use in mechanically ventilated SAH patients [108].

The TAME trial is a large multicentre phase III trial that is underway to determine whether a therapeutic mild HC improves neurological outcome at six months compared to standard care (normocapnia), in resuscitated cardiac arrest patients who are admitted to the ICU (NCT03114033). The main objective of this trial will be to determine whether targeted therapeutic mild HC (PaCO_2_ 50–55 mmHg) during the first 24 h of mechanical ventilation in the ICU improves neurological outcome at 6 months compared to standard care (normocapnia, PaCO_2_ 35–45 mm Hg). The phase II arm of this study reported safety and feasibility of using mild HC in cardiac arrest patients [109].

The effect of mild permissive HC (approx. 48 mmHg PaCO_2_) on cerebral perfusion in infants was examined in a study titled ‘Assessment of Cerebral Vasoreactivity Using Near-infrared Spectroscopy (NIRS) in Infants (VARO)’ (NCT02429154) [110]. This study aimed to use HC to counteract the interference of ventilation with cerebral perfusion due to changes in thoracic pressures or hypocapnia leading to vasoconstriction. Although the study is completed, trial results have not been reported by the authors aside from conference proceedings in which they describe that HC prevents the mild reduction in tissue haemoglobin index and in mean arterial pressure observed in normocapnia group. They concluded that arterial pressure is an important component of cerebral oxygenation in moderate HC.

## 9. Pre-term infants

There have been some small clinical trials in neonates and the young paediatric setting demonstrating at least a lack of harm and suggestions of benefit [111,112]. Late Permissive Hypercapnia for Intubated and Ventilated Preterm Infants (HYFIVE, NCT02799875) is currently recruiting participants for a study of the use of pH controlled late permissive HC. Inclusion criteria for the study are: term neonates during infancy and for elective surgery requiring general anaesthesia and endotracheal intubation. Higher (arterial PaCO_2_ 60–75 mmHg, pH ≥ 7.20) and lower permissive HCA (arterial PaCO_2_ 40–55 mmHg, pH ≥ 7.25) levels are being investigated in preterm infants. Outcomes measured are alive VFD, all causes of death, BPD, post-natal steroid treatment for BPD.

## 10. Extracorporeal CO_2_ removal

Extracorporeal CO_2_ removal (ECCO_2_R) devices pump blood through an artificial circuit incorporating a gas exchanger with a large diffusing capacity that removes CO_2_ directly from the bloodstream independent of the lungs. ECCO_2_R is used to facilitate ultra-low tidal volume protective lung ventilation, as it allows the tidal and minute volumes to be further reduced while preventing excessive accumulation of CO_2_. Small studies have shown that ECCO_2_R does enable very low tidal volume ventilation strategies to be used in ARDS patients without extreme levels of acidosis, which may be effective in reducing lung injury [113] and to be safe in severe ARDS [114,115].

As of 2018, there have been two randomized control trials evaluating extracorporeal CO_2_ removal in ARDS [116], with one study [117] finding no significant difference in mortality but a dramatic increase in the complexity of care and as such was not a recommended treatment. The second study was performed by Bein *et al*. [114] who showed that ECCO_2_R did not confer any improved survival but allowed for lower tidal volumes reducing the risk of VILI.

Richard *et al*. [118] show that in the treatment of ARDS, ultra-low tidal volume ventilation of VT ml kg^−1^ without ECCO_2_R is a potential option for severe ARDS patients, maintaining a similar hospital mortality rate when compared to a previous observational study using ECCO_2_R [119]. However, this also came with an increased severity, higher respiratory rate and severe acidosis (pH < 7.2) in a portion of the patients (33%) [118]. These studies highlight that an increased PaCO_2_ is well tolerated when considering the potential morbidity/mortality associated with an increased tidal volume; an observational study performed by Needham *et al*. [120] showed an initial increase of 1 ml kg^−1^ leads to a 23% higher mortality rate.

## 11. Conclusion and future directions

As it stands, there is much work to be done about the potential use of HC or HCA, but many reasons to be optimistic about both avenues of basic scientific investigation and emerging therapeutic options. The complex mechanisms of HC need to be tightly regulated to provide true benefit. There is also a gradual realization that, while pH effects may be non-specific, they are not exclusively deleterious, and may have a role to play in a strictly controlled medical environment. Of greater excitement is the emerging possibility of specifically targeting molecules that are under the control of CO_2_, perhaps through utilization of small molecules that reproduce similar binding and influence. Candidate targets include aquaporins, soluble adenyl cyclases and various constituents of the NF*κ*B and other pathways. This ability to have the best of both worlds, an enhancement of the beneficial effects of HC without its often simultaneous negative consequences, would be a significant boon in therapeutics for the critically ill and inflammation in general. Revolutionary approaches such a direct control of blood CO_2_ concentration will allow fine tuning of this new therapeutic paradigm. The future is indeed bright for this once maligned little ‘waste’ molecule.

## Data accessibility

This article has no additional data.
